# Prevalence of Hearing Impairment Among High-Risk Newborns in Ibadan, Nigeria

**DOI:** 10.3389/fped.2018.00194

**Published:** 2018-07-16

**Authors:** Adeyemi A. Labaeka, Olukemi O. Tongo, Babatunde O. Ogunbosi, James A. Fasunla

**Affiliations:** ^1^Department of Paediatrics, University College Hospital Ibadan, Ibadan, Nigeria; ^2^Department of Paediatrics, University of Ibadan, Ibadan, Nigeria; ^3^Department of Otorhinolaryngology, University College Hospital Ibadan, Ibadan, Nigeria

**Keywords:** high-risk newborn, hearing impairment, auditory brainstem response (ABR), sensorineural hearing loss, Nigeria

## Abstract

The burden of severe hearing impairment is increasing with two-thirds of these hearing impaired people residing in developing countries. Newborn hearing screening helps to identify early, babies who need intervention in order to prevent future disability. Neither universal nor targeted hearing screening programme is available in Nigeria.

**Objectives:** This study was carried out to assess the prevalence of hearing impairment among high-risk newborns in UCH and the associated risk factors.

**Materials and Methods:** Two hundred one newborns in the neonatal unit of UCH with risk factors for hearing impairment had hearing screening done using automated auditory brainstem response (AABR) at 30, 45, and 70 dB at admission and discharge, and those that failed screening at discharge were rescreened at 6 weeks post-discharge.

**Results:** Eighty-three (41.3%) and 32 (15.9%) high-risk newborns failed at admission and discharge screening respectively, and 19 (9.5%) still failed at follow up screening. The majority of hearing loss at follow up was bilateral (94.7%) and severe (52.6%). The risk factors associated with persistent hearing loss at follow up were acute bilirubin encephalopathy (RR = 11.2, CI: 1.4–90.6), IVH (RR = 8.8, CI: 1.1–71.8), meningitis (RR = 4.8, CI: 1.01–29), recurrent apnoea (RR = 2.7, CI: 1.01–7.3), severe perinatal asphyxia NNE III (RR = 7, CI: 2.4–20.2).

**Conclusion:** Severe and bilateral hearing impairment is a common complication among high risk newborns in UCH persisting till 6 weeks post-neonatal care. Severe perinatal asphyxia with NNE III, ABE, IVH, meningitis and administration of amikacin for more than 5 days were significant risk factors. We recommend that SCBU graduates with these risk factors should have mandatory audiologic evaluation at discharge.

## Introduction

Intact hearing is an essential requirement for speech and language development, so children with hearing loss will be unable to develop speech and this puts such children at a disadvantage socially, emotionally, educationally, and economically among their peers (1, 2) Hearing impairment is a common treatable disability in childhood if diagnosed early and appropriate intervention instituted. The Joint Committee on Infant Hearing (JCIH) of American Academy of Paediatrics (AAP) defines target hearing loss as “congenital permanent bilateral, unilateral, sensory, permanent conductive, or neural hearing loss (auditory neuropathy/dyssynchrony), averaging 30–40 dB or more in the frequency region important for speech recognition (~500–4,000 hertz),” which will interfere with the normal development of speech and language (1, 3).

The prevalence of hearing impairment has been on an astronomical increase especially in the last three decades. World Health Organisation (WHO) reported 42 million people affected globally in 1985, which has increased to 360 million people by the year 2012 (4, 5). Globally, about 796,000 babies suffer permanent hearing loss within the neonatal period annually and majority of these newborns reside in developing countries where routine hearing screening is not readily available (5, 6). Studies have shown that children who had Early Hearing Detection and Intervention system (EHDI) before 6 months of age achieved higher vocabulary, articulation, cognitive, social and emotional development than those who has the same interventions but later (1, 7–9). A retrospective review of 6 years of Universal hearing screening in Qatar, showed that 95% coverage of all babies born was achievable which enabled identification of up to two-thirds of babies with hearing impairment by 6 months of age. This identification made it possible to offer interventions to majority of them by 2.5 years of age (10).

The tests of auditory function recommended for use in newborns are the otoacoustic emmission tests (OAEs) and automated auditory brain stem response (AABR). These two methods provide non-invasive recordings of the physiologic activities of the auditory system and also require minimal patient cooperation. Both technologies are affected by fluids and debris in the auditory canal in the first few days of life. AABR reflects the integrity of the entire auditory pathway, while OAEs will only assess the peripheral auditory system (4). A sensitivity of 85–100% and specificity of 91–95% have been reported for OAEs. The automated auditory brain stem response is recommended for use in NICU graduates who have stayed up to 5 days on admission. Two stage screening tests utilizing TOAE and then AABR have been used in large screening programmes to avoid false failed or passed results.

The actual prevalence of hearing impairment in Nigeria is unknown but Olusanya, Wirz, and Luxon in a community-based infant hearing screening programme using a two-stage screening protocol (TOAE and AABR), reported an incidence of permanent hearing impairment (≥30 dB hearing level) among infants attending immunization clinics most of whom were born outside regular hospitals as 28 per 1,000 babies screened (11). This prevalence is much higher than the 1.5 per 1,000 obtained from a similar community based study in South Africa (12). This very high prevalence was acknowledged to be the highest reported in the world but not explained.

Most children with hearing impairment in Nigeria are diagnosed only when speech has failed to develop, partly because of the absence of routine or targeted newborn hearing screening (13, 14) and parents' failure to or delay in recognizing the problem (15). The JCIH recommends routine hearing screening on all newborn babies, however, for developing nations and remote areas where lack of resources might limit the development of newborn screening programmes, an initial focus on screening of high-risk newborns and NICU graduates is recommended (1). Screening in this group is important as the risk of moderate to severe permanent hearing loss is 10–20 times higher in high-risk newborns than in the general population (16). However, even this is hampered by constraints of manpower and availability and/or access to screening modalities and probably non-recognition of the need by the appropriate health authorities, consequently, newborn screening programmes are not available in many developing countries including Nigeria. Documenting the burden of this problem and the risk factors in such resource-limited settings should help in planning such screening programmes, assist in prioritizing allocation of limited resources and also guide institution of measures to limit exposure to these risk factors. In addition, it will guide the development of guidelines for proper follow up of this group of newborns at risk of hearing impairment. Most studies on newborn hearing impairment in Nigeria has been fraught with methodological limitations with the risk factors involved not clearly defined or inferred from historical recall which may be misleading (11, 13, 14, 17).

This study set out to perform hearing screening in all neonates admitted into the neonatal unit of the University College hospital with risk factors for hearing impairment pre and post exposure to the risk factors. This was with a view to defining the prevalence of hearing impairment in high risk neonates and determine the associated risk factors.

## Materials and methods

This was a longitudinal cohort study of babies with risk factors (JCIH risk factors) (1) for hearing impairment admitted into the neonatal unit of the University College Hospital (UCH), Ibadan, Nigeria, between November 2014 and February 2015. Newborns whose parents gave written informed consent were enrolled into the study. All enrolled neonates were screened for hearing impairment with an automated auditory brainstem response (AABR) machine (Natus Algo ® 2e) at admission, discharge and 6 weeks after discharge. Babies with craniofacial abnormalities or who already had more than 24 h exposure to ototoxic medications before admission were excluded from the study. The screening was done in a quiet room while baby was calm or sleeping, both ears were screened simultaneously at 35, 40, and 70 dB frequencies. For the purpose of this study, the degree of hearing loss was classified as mild (35–40 dB HL), moderate (41–70 dB HL) and severe (>70 dB HL). The screener automatically displayed “pass” when it had collected sufficient data to establish with 99.96% statistical confidence that an ABR signal was present and consistent with the template at a minimum of 1,000 sweeps when screening at 35 dB click, and a minimum of 2,000 sweeps when screening at 40 dB and 70 dB clicks. “Refer” result was displayed when it did not establish with 99.96% statistical confidence that the ABR signal was present at 15,000 sweeps when screening at 35 and 40 dB clicks, or at 10,000 sweeps when screening at 70 dB click. In babies with neonatal jaundice, acute bilirubin encephalopathy was diagnosed using clinical Bilirubin-Induced Neurologic Dysfunction (BIND) score (18). Post asphyxia encephalopathy was graded into stages I, II, and III according to the Sarnat and Sarnat classification.

Data was entered and analyzed using SPSS version 20.0 software. Descriptive statistics such as means, standard deviations and medians (ranges) were used to report continuous variables while categorical variables were summarized by percentages. Pearson Chi-square test was utilized for bivariate analysis of dependent and independent categorical variables (risk factors associated with hearing impairment), while student *t*-test was used to compare the mean TSB of subjects with hearing impairment and those with normal hearing. Relative risk was calculated for each risk factor at 95% confidence interval. Logistic regression analysis was utilized for multivariate analysis to determine predictors of hearing and statistical significance was set at 5%.

The calculated minimum sample size required at 95% confidence interval was 200 subjects.

Ethical approval for the study protocol was obtained from the University of Ibadan/ University College Hospital Joint Ethics Review Committee (UI/EC/13/0355).

## Results

**Figure 1:**
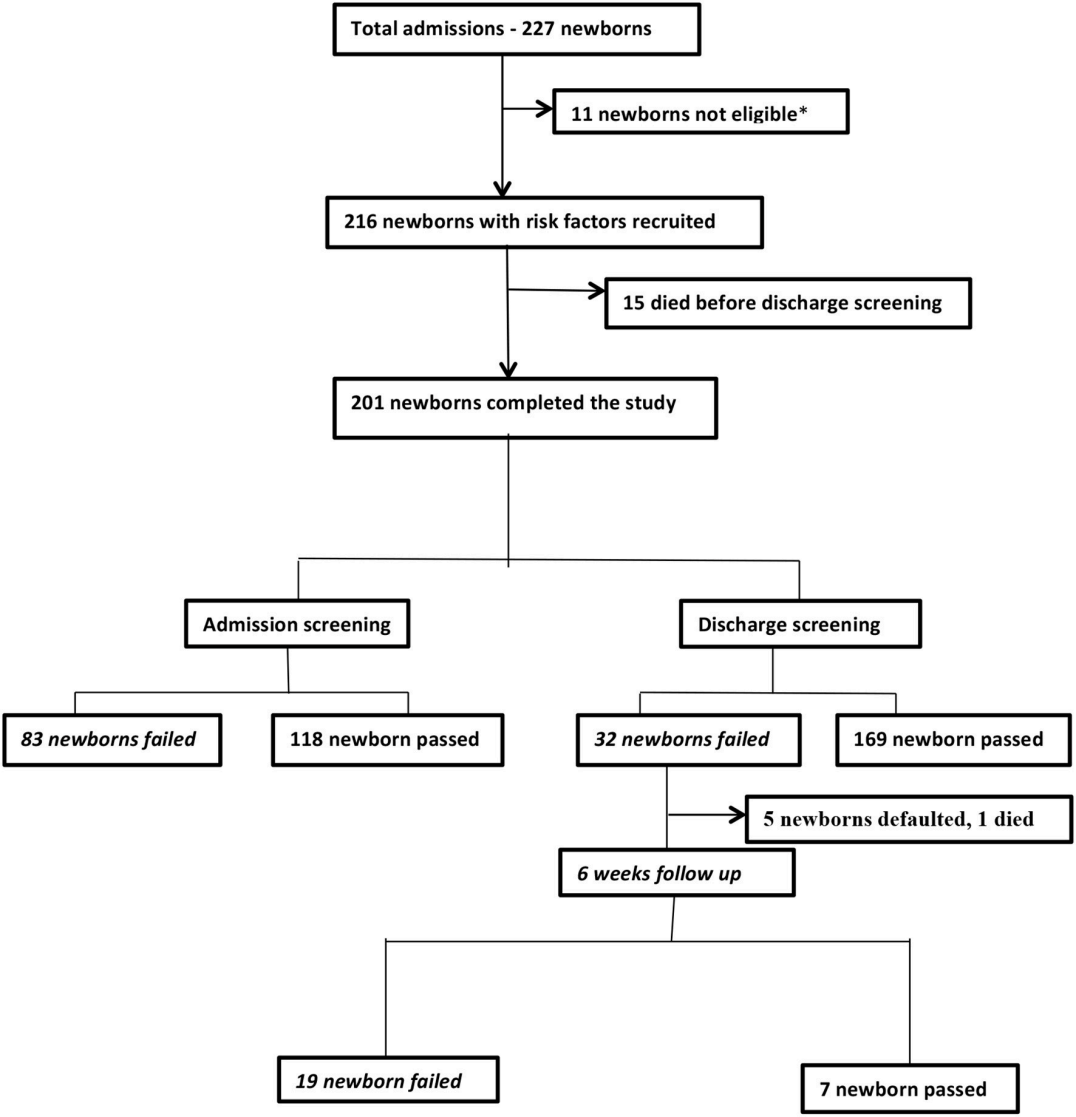
Parents of three newborns did not give consent, two newborns had no risk factors another one had maxillofacial malformation, while five newborns had been on multiple daily doses of intravenous aminoglycosides for more than 24 h prior to admission in our facility.

During the study period, 227 newborns were admitted into the neonatal unit, 216 were recruited into the study but only 201 (93%) completed the study as shown in Figure 1 below. The 201 newborns, comprised of 112 (55.7%) males and 89 (44.3%) females, giving a male: female ratio of 1.3:1. The median age at admission was 6 h (range of 0.5–672 h), but the median age at admission of newborns delivered in and outside UCH was 3 and 37 h respectively, and the mean gestational age was 35.7 ± 3.9 weeks (range of 28–41 weeks). The majority of the newborns were term (*n* = 106, 52.7%) while 95 (47.3%) were preterm. The mean ± SD weight on admission was 2350 ± 910 g (male 2,500 ± 880 g, female 2,160 ± 920 g), mean length was 45.0 ± 5.5 cm and mean occipito-frontal circumference was 32.1 ±3.4 cm. Birth weight was ≥ 2,500 g in 97 (48.3%), 1,500– < 2,500 g in 60 (29.9%) babies., 1,000– < 1,500 g in 36 (17.9%) babies and < 1,000 g in 8 (4%) babies. Five (2.5%) were large for gestational age (LGA) while 31 (15.4%) were small for gestational age (SGA) and the rest (*n* = 165, 82.1%) were appropriate for gestational age (AGA).

Almost half of the subjects (*n* = 95, 47.3%) were delivered in UCH, the rest were outborn. Neonatal sepsis was the most prevalent risk factor for hearing loss in 189 (94%) of the newborns, followed by amikacin administration, hyperbilirubinaemia, and severe perinatal asphyxia. Other risk factors for hearing loss among the subjects are as shown in Table 1 below. Only one of the subjects had exposure to a single risk factor, others had multiple risk factors with 26.4% having as many as >5 risk factors. Hyperbilirubinaemia necessitating, at least, phototherapy was documented in 89 (44.3%) newborns, exchange blood transfusion in 11 (5.5%) and acute bilirubin encephalopathy (ABE) was diagnosed in 5 (2.5%) of the subjects. The mean ± SD peak TSB for all newborns with hyperbilirubinaemia was 12.0 ± 7.3 mg/dl, while it was 35.5 ± 4.6 mg/dl in those with ABE and 10.9 ± 5.3 mg/dl in those without ABE. None of the subjects had mechanical ventilation. The median number of episodes of apnoea was 2 with a range of 2–20 episodes.

**Table 1:** Risk factors for hearing impairment among the subjects.

**Risk factors**	***n* (%) *N* = 201**
Neonatal sepsis	189 (94)
Hyperbilirubinaemia	89 (44.3)
Severe perinatal asphyxia	82 (40.8)
No neonatal encephalopathy	13 (6.5)
Neonatal encephalopathy 1	17 (8.4)
Neonatal encephalopathy II	46 (23)
Neonatal encephalopathy III	6 (3)
Prematurity < 34 weeks	73 (36.3)
Amikacin therapy > 5 days	69 (34.3)
Very Low birth weight	43 (21.4)
Respiratory distress syndrome	35 (17.4)
Small for gestational age	35 (17.4)
One minute APGAR score ≤ 3	31 (15.4)
Recurrent Apnoea	25 (12.4)
Five minute APGAR score ≤ 5	15 (7.5)
Frusemide administration	13 (6.5)
Gentamycin therapy > 7 days	13 (6.5)
Intraventricular hemorrhage	11(5.5)
Vancomycin therapy	8 (4.0)
Meningitis	5 (2.5)
Acute bilirubin encephalopathy	5 (2.5)
Dysmorphic features	3 (1.5)
Intrauterine infection	2 (1.0)
Family history of SNHL	1 (0.5)

*Encephalopathy was graded using the Sanart and Sanart classification SNHL, sensorineural hearing loss*.

The mean duration of aminoglycoside usage was 7.3 ± 4.3 days. The mean ± SD daily doses of the ototoxic drugs used were 1.1 ± 0.3, 14.9 ± 0.6, 5.0 ± 0.0 and 18.3 ± 5.2 mg/kg/day for frusemide, amikacin, gentamycin and vancomycin respectively, while the mean duration of exposure to ototoxic drugs was 1.7 ± 1.3, 5.7 ± 2.1, 7.3 ± 2.4 and 7.0 ± 2.7 days respectively for the above-listed drugs. The median duration of hospital stay was 15 days and a range of 4–55 days. Eighty three newborns (41%) failed at admission screening and 32 (15.9%) failed on discharge. Of these 32, 26 presented for follow up, 1 had died at home and 5 (15.6%) were lost to follow up. Among the 26 that presented 19 (9.5%) still failed screening. Table 2 shows the types of hearing impairment during each stage of hearing screening. There was no additional newborn who failed AABR screening at discharge that had not previously failed on admission. Table 3 shows the degree of impairment in the newborns who failed AABR screening at the different periods, severity of hearing impairment was noticed to improve during the course of treatment.

**Table 2:** Types of hearing impairment.

**Types of hearing impairment**	**On admission *n* (%)**	**On discharge *n* (%)**	**At follow up *n* (%)**
**Time of screening**			
Unilateral	15 (18.1)	4 (12.5)	1 (5.3)
Bilateral	68 (81.9)	28 (87.5)	18 (94.7)
Total	83 (100)	32 (100)	19 (100)

**Table 3:** Severity of hearing impairment at different stages of screening.

**Screening frequencies (dB)**	**Number of subjects that failed (%)**		
	**On admission**	**On discharge**	**At follow up**
35 (mild)	38 (45.8)	14 (43.8)	6 (31.6)
40 (moderate)	19 (22.9)	4 (12.5)	3 (15.7)
70 (severe)	26 (31.3)	14 (43.8)	10 (52.6)
Total	83 (100)	32 (100)	19 (100)

## Risk factors associated with hearing impairment

ABE and severe perinatal asphyxia were significantly associated with failed AABR sreening while ABE had the greatest risk of failed screening during admission screening as shown in Table 4.

**Table 4:** Risk factors associated with hearing impairment during admission, discharge, and follow up screening.

**Risk factor**	**Subjects *n* (%)**	**Failed *n* (%)**	**RR**	**95% CI**	***P*-value**
**ADMISSION AABR SCREENING** ***N*** = **201**					
Acute bilirubin encephalopathy	5 (2.5)	4 (2.0)	2.5	1.9–3.4	**0.007**
Very low birth weight	43 (21.4)	23 (11.4)	1.3	0.9–1.8	0.067
Prematurity GA < 34 weeks	73 (36.3)	33 (16.4)	1.02	0.8–1.4	0.395
Intrauterine infection	2(1)	1(0.5)	1.2	0.3–4.8	0.802
Meningitis	5 (2.5)	4 (2)	1.9	0.2–17	0.075
Severe perinatal asphyxia	82 (40.8)	42 (20.9)	1.3	0.8–2.3	**0.018**
NNE I	17 (8.5)	8 (4)	1.2	0.4–3.3	
NNE II	46 (22.9)	24 (11.9)	1.4	0.7–2.7	
NNE III	6 (3)	0 (0)	2.5	1.1–5.8	
**DISCHARGE AABR SCREENING** ***N*** = **201**					
Acute bilirubin encephalopathy	5 (2.5)	4 (2.0)	7.3	5.2–10.3	0.000
Meningitis	5 (2.5)	4 (2.0)	4.5	1.8–40.6	0.000
Vancomycin therapy	8 (4.0)	5 (2.5)	3.5	1.1–15.4	0.000
IVH	11 (5.5)	10 (5.0)	6.7	3.3–13.5	0.000
Recurrent Apnoea	25 (12.4)	11 (5.5)	2.7	1.1–6.5	0.000
Severe perinatal asphyxia NNE III	6 (3)	0(0)	7.5	3.0–18.4	0.018
Amikacin > 5 days	69 (34.3)	18 (9)	2.5	1.2–5.4	0.02
**FOLLOW UP AABR SCREENING** ***N*** = **26**					
Acute bilirubin encephalopathy	2 (7.7)	2 (100)	11.2	1.4–90.6	0.003
Meningitis	4 (15.4)	3 (75)	4.8	1.01–29	0.007
Vancomycin therapy	5 (19.2)	3 (60)	3.0	0.7–13.3	0.032
Recurrent Apnoea	8 (30.8)	7 (87.5)	2.7	1.01–7.3	0.002
Intraventricular hemorrhage	9 (34.6)	8 (88.8)	8.8	1.1–71.4	0.000
Severe Perinatal Asphyxia NNE III	4 (2.0)	4 (100)	7.0	2.4–20.2	0.000

Eleven babies presented with TSB >20 mg/dl of which 5 had acute bilirubin encephalopathy. Nine of the 11 failed admission screening. At discharge screening, 1 of these 9 had passed, 2 move from severe to mild impairment. Of the 8 that still had impairment at discharge, 1 died at home, 1 was lost to follow up, and 1 moved from mild impairment to pass at follow up screening, and 5 still failed at follow up.

Newborns with neonatal encephalopathy stage III from severe asphyxia were 7.5 times more at risk of failing AABR screening than those without asphyxia. Stages I and II encephalopathy were not significantly associated with failed AABR screening. Intraventricular hemorrhage (IVH), ABE, meningitis, recurrent apnoea, vancomycin administration and administration of amikacin for longer than 5 days were significantly associated with the risk of failed AABR screening during the discharge screening as shown in Table 4. Severe perinatal asphyxia NNE III, ABE, IVH, recurrent apnoea and meningitis were also significantly associated with the risk of failed screening during follow up screening. ABE had the greatest risk of failed hearing screening during follow up screening as shown in Table 4. Those who failed AABR screening on admission, at discharge and at follow up, had significantly higher mean TSB as shown in Table 5 below.

**Table 5:** Mean TSB among babies who passed compared with those who failed AABR at different stages of screening.

**Time of screening**	**TSB Mean** ± **SD**		***P*-value**
	**Failed**	**Passed**	
Admission screening	13.6 ± 9.7	10.7 ± 4.8	0.045
Discharge screening	20.9 ± 12.6	10.5 ± 4.8	< 0.000
Follow up screening	25.4 ± 13.0	11.1 ± 5.9	< 0.000

## Discussion

The effect of hearing impairment on the newborn, the family and the community is lifelong if intervention is not sought early in life. Early diagnosis and appropriate and timely intervention remain the only way to mitigate its effect on speech, language and cognitive development (1, 2). The prevalence of failed hearing screening using the AABR among high-risk newborns in this study progressively reduced from 41.3% on admission, 15.9% at discharge to 9.5% at follow up. There were no new cases of failed hearing screening noted in the study population after exposure to treatment in the unit apart from those present at admission. This implies that all cases who failed the screening were already present at presentation even as early as 24 h of life in some cases.

The high prevalence of failed result during admission screening may be partly due to the presence of fluid and debris in the middle ear expected during the first 48 h of life. This is why multistage screening is advisable and as seen in this study, the prevalence of failed results reduced with time. Though not all the subjects presented within the first 48 h of life, hence failed screening in those ones may be due to the risk factors present in them, such was the case with newborns with severe hyprbilirubinaemia in whom 9 out of 11 failed on admission, 8 at discharge and 5 still failed at follow up. Similar result was reported by Lasisi et al in a pilot Newborn hearing screening programme in a rural/sub-urban community in Nigeria, in which a prevalence of failed AABR was 49.4% in the first screening (as seen in Table 6) which was done in the first 72 h of life (14). The overall prevalence of failed AABR in Special Care Baby Unit (SCBU) graduates on discharge of 15.9% in this study is similar to the 13.5% reported by Hee-Joung et al. in Korea (19) but much higher than 8.5% reported by Akinola et al. (16) at the same center using transient evoked otoacoustic emission (TEOAE). but their study population included normal babies with low risk and SCBU graduates. The similarities with the Korean study may be a result of similar methodology i.e., AABR and similar study participants.

**Table 6:** Comparison of the findings in the present study with previous studies.

	**Author**	**Year**	**Country**	**Technology**	**Study population**	**Result**
1.	Labaeka et al (present study)	2018	Nigeria	AABR	201 high risk neonates	1st screening: 41.3% 2nd screening: 15.9% 3rd screening: 9.5% Risk Factors: ABE, IVH, SPA NNEIII, meningitis, vancomycin, amikacin administration > 5 days, recurrent apnoea.
2.	(17)	2014	Nigeria	OAE	386 neonates	1st screening : 29% failed, 2nd/discharge screening: 8.5% Risk factor; Prematurity
3.	(14)	2014	Nigeria	AABR	453 neonates	1st screening = 49.4%, 82.5% lost to follow up, 14/40 passed at follow up screening Risk factors: maternal pre-eclampsia
4.	(19)	2006	South Korea	ABR	474 high risk newborn	Discharge screening: 13.5%, Risk factor; Neonatal jaundice, VLBW, LBW, perinatal asphyxia, amikacin administration for > 15 days

SCBU graduates have been previously reported to be at high risk of sensorineural hearing loss (SNHL) either from their conditions or interventions received (20). Though all the babies who failed at discharge and follow up had already failed AABR on admission, it is essential to minimize further exposure to risk factors such as medications and other interventions while being cared for in the SCBU/NICU. The prevalence of persistent failed hearing screening at follow up in this study (23% of those who failed on admission) was slightly lower than the 25.6% reported by Min Young et al in Korea (21) while the corresponding figure reported by Akinola et al. (16) was 29.3%. The initial high prevalence is thought to be related to the very early age at the initial screening in a large proportion of the participants during which the presence of amniotic fluid and debris in the middle ear canal may have contributed significantly to the initial failed screening but improved with time as this cleared as is naturally expected.

Overall, about a tenth of the high-risk newborns treated in the newborn unit of UCH still failed screening at follow up 6 weeks after discharge, this is higher than 4.4 and 8.5% reported by Olusanya and Akinola respectively (17, 22) This is a significant proportion of SCBU graduates and if not identified early and interventions offered, may contribute to the long-term disability associated with neonatal intensive care with its attendant contribution to the burden of care especially in an low and middle-income countries (LMIC) like Nigeria. In a study in Kuwait among similar high risk neonates, a much higher proportion (46%) failed screening at discharge though they used diagnostic ABR and TEOAE. In addition, the mean gestational age of their subjects was much lower than ours though it is not clear to what extent this might have affected their result (23). However, another similar study by Maqbool M et al. in India, got a similar prevalence of 16% at discharge and 10% at follow up (24) while Hee-Joung (19) in Korea reported 13.5% on discharge. These are much higher than the 3.8% among NICU graduates with risk factors reported by Kong et al also in Korea though they excluded preterms less than 36 weeks and birthweight less than 2,200 g (25). In situations like ours where routine newborn hearing screening is not available, it is expedient to have a screening programme for these high-risk babies if the gains of providing intensive care in the first place are to be maximized in order for them not to have limitations in contributing their quota to national productivity in future. In view of the rate loss to follow up of 15.6% and the high prevalence of false failed screening on admission, the period of discharge might be the optimal time of screening of high risk neonates in our setting in order to identify as many cases as early as possible. Repeat screening after 6 weeks is also recommended for those who fail the discharge screening in order to identify those who will improve while not delaying further evaluation and intervention in those who will have persistent impairment. This timing is supported by the findings of Apuzzo and Yoshinaga-Itano who showed that newborns with hearing impairment identified before 2 months of age had significantly higher scores in general development and expressive language than those identified later despite having similar interventions (26).

The multiple screening as seen in this study reduces the likelihood of false failed screening as demonstrated by Mishra (27). Multiple screening also allows resolution of clinical conditions such as perinatal asphyxia, prematurity, hyperbilirubinaemia and presumed neonatal sepsis (28). In addition, some previously undetected cases of hearing loss that might manifest later could be identified by the multiple screening protocols as reported by Jaideep Bhatt et al in India and Min-Young et al in Korea (21, 28). In the present study, none of the babies who had previously passed on admission failed at discharge.

The risk factors associated with hearing impairment in our study were similar to the findings of Martinez-Cruz et al. (29) who reported NNJ requiring EBT, IVH, meningitis, bronchopulmonary dysplasia, frusemide, and amikacin as predictors of hearing impairment. There was a relatively low frequency of usage of frusemide (6.5%) and a small proportion of extreme prematurity in our study and this may be the reason why they were not found to be significant risk factors. This is in contrast to the findings of Akinola (17) and Hee-Joung (19) who reported prematurity as risk factors.

In view of the multiplicity of risk factors in some cases and the fact that exact causal relationships between some of the identified risk factors and hearing impairment could not be established with certainty, it is recommended that all these factors be taken into consideration in the management of these newborns especially as it relates to ototoxic medications. For instance, dosage modifications in the presence of other clinical risk factors particularly in settings where drug levels cannot be monitored.

The presence of stage III encephalopathy has previously been identified as a definitive marker of hearing loss in asphyxiated infants and its tendency for persistent abnormal AABR in the newborn (30, 31). This was also corroborated in this study. ABE had the second highest relative risk in this study for causing failed hearing screening during the discharge screening and newborns with NNJ and failed screening had much higher mean TSB than those with NNJ but passed screening. This further supports the report of a systemic review which showed that the higher the total peak serum bilirubin, the greater the risk of hearing abnormality (32). Wickremasinghe et al. (33) in a nested double cohort study involving 525,409 infants born ≥ 35 weeks gestation, reported that bilirubin level > 35 mg/dl were associated with statistically significant increased risk of SNHL. The mean TSB in newborns with ABE in this study was > 35 mg/dl. The significance of this is that every effort should be put into preventing development of such severe jaundice.

The relative of risk of failed screening due to meningitis, IVH and amikacin in this study is similar to what was reported by Martinez-Cruz et al in their evaluation of high-risk newborns in another developing country (29).

Aminoglycoside induced ototoxicity in newborns has been documented by several authors, (19, 20, 29, 34–37) it causes a dose-dependent outer hair cell loss by inducing apoptosis and this risk is further heightened in the presence of renal impairment. Intracellular accumulation of aminoglycoside occurs through the megalin-independent cation influx and the influx maybe potentiated by ambient noise and loop diuretics. In the review by Smit et al, it was documented that once daily dose of amikacin at 15 mg/kg/24 h is less likely to be associated with SNHL (34). The aminoglycoside induced hearing impairment in this study may therefore be a synergism of drug exposure, environmental noise and other associated risk factors (4). This further underscores the need for individualized dosing aminoglycosides in the management of newborn considering the weight, postnatal age, perinatal asphyxia, hypothermia, and ibuprofen use as suggested by Smit et al. (34) and Cristea et al. (35).

In conclusion, there is a high prevalence of hearing impairment persisting till 6 weeks post-neonatal care in high-risk newborns in UCH, the majority of which are severe and bilateral. Severe perinatal asphyxia with NNE III, ABE, IVH, meningitis and administration of amikacin for more than 5 days were significant risk factors. Based on the fact that hearing impairment is of public health concern and is a remediable condition when detected early, hearing screening is cost effective but not widely available in resource limited settings such as ours and on the basis of our findings, we recommend that SCBU graduates with stage III encephalopathy, acute bilirubin encephalopathy, intraventricular hemorrhage, meningitis and amikacin therapy for more than 5 days should have mandatory audiologic evaluation at discharge. This should be incorporated into the management protocols of newborn units pending widespread availability of facilities for routine UNHS. The ultimate is to have a universal hearing screening programmes combining TOAE and AABR in order to identify babies with auditory neuropathy as well as those with cochlea abnormalities.

## Ethics statement

This study was carried out in accordance with the recommendations of The National Code for Health Research Ethics, University of Ibadan/University College Hospital Ethics Committee with written informed consent for all subjects. All subjects gave written informed consent in accordance with the Declaration of Helsinki. The protocol was approved by University of Ibadan/University College Hospital Ethics Committee.

## Author contributions

LA: Conceptualization and design of the work, drafting of proposal, data collection, analysis and interpretation, drafting of manuscript for publication; TO: Conceptualization and design of the work, drafting and revision of proposal, data analysis and interpretation, drafting and revision of manuscript for publication; BO: drafting of proposal, data interpretation, revision of manuscript for publication; FJ design of the work, revision of proposal, data interpretation, revision of manuscript for publication.

### Conflict of interest statement

The authors declare that the research was conducted in the absence of any commercial or financial relationships that could be construed as a potential conflict of interest.
